# Strain differences in proliferation of progenitor cells in the dentate gyrus of the adult rat and the response to fluoxetine are dependent on corticosterone

**DOI:** 10.1016/j.neuroscience.2008.08.072

**Published:** 2008-12-02

**Authors:** S. AlAhmed, J. Herbert

**Affiliations:** Department of Physiology, Development and Neuroscience, and Cambridge Centre for Brain Repair, University of Cambridge, Downing Street, Cambridge CB2 3DY, UK

**Keywords:** progenitor cells, neurogenesis, hippocampus, strain differences, corticoids, AADC, aromatic amino acid decarboxylase, ADX, adrenalectomized/adrenalectomy, ANOVA, analysis of variance, CT, circadian time, HPA, hypothamalo-pituitary–adrenal, IHC, immunohistochemistry, KPBS, potassium phosphate buffer

## Abstract

This paper investigates the role of differences in adrenal cortical function on the proliferation rate of progenitor cells in the dentate gyrus of the hippocampus in adult Sprague–Dawley (SD) and Lister-Hooded (LH) male rats. SD rats had around 60% more cells labeled with Ki67 (an index of mitosis) than LH rats under basal conditions. Bilateral adrenalectomy (ADX) increased levels in both strains, but by unequal amounts, such that post-ADX numbers of Ki67-labeled cells were similar in both strains. Daily injections of 5 mg/kg corticosterone for 7 days reduced levels to similar values in ADX rats of both strains. The activity of progenitor cells in either strain did not respond to daily i.p. injections of fluoxetine (10 mg/kg) for 14 days, but an equivalent dose administered by osmotic minipump stimulated proliferation in both by a similar proportional amount, such that strain differences persisted. S.c. implantation of a corticosterone pellet (75 mg), which flattens the diurnal rhythm in corticosterone, prevented fluoxetine delivered by minipump from activating progenitor cell mitosis in SD rats, as it had in the LH strain in a previous study. These results show that much, if not all, of the marked strain differences between SD and LH rats in progenitor cell activity, and hence rates of neurogenesis in the dentate gyrus may be ascribed to corresponding differences in adrenal cortical activity.

Neurogenesis, or the generation of new neurons, in the dentate gyrus of the hippocampus is now known to persist during adulthood in several mammalian species (Eriksson et al., 1998; Gage, 2000; Gould et al., 1999, 2000; Yamashima et al., 2007). Far from being constant, the rate of neurogenesis is regulated by many factors (Bruel-Jungerman et al., 2007; Duman, 2005; Engesser-Cesar et al., 2007; Kuhn et al., 1997; Levenson and Rich, 2007; Luo et al., 2007; McEwen, 2002; Wong and Herbert, 2005), among which glucocorticoids are prominent (Cameron and Gould, 1994; Huang and Herbert, 2006; Joels and Vreugdenhil, 1998; Wong and Herbert, 2005, 2006). Neurogenesis is exquisitely sensitive to these steroids, though whether this is because of direct action on the progenitor cells themselves, or an indirect one on other cells forming the cellular niche within which neurogenesis occurs is still uncertain. Corticosterone treatment of rats or mice sharply reduces the proliferative rate of progenitor cells (Cameron and Gould, 1994; Gould et al., 1992; Wong and Herbert, 2004), as well as their ability to form mature neurons (Wong and Herbert, 2006). Adrenalectomy (ADX) has the reverse effects, showing that endogenous corticoids also regulate neurogenesis (Gould et al., 1992; Cameron and Gould, 1994). The extreme sensitivity to corticoids is further reflected by the diurnal rhythm in corticosterone being able to drive a corresponding rhythm in the progenitor cells (Pinnock et al., 2006).

In addition to its proximate control of neurogenesis, the diurnal rhythm of corticosterone in the rat has another function: it gates the access of other agents, such as drugs that alter either 5-HT (e.g. fluoxetine) or nitric oxide (NO) (e.g. NG-nitro-L-arginine-methyl-ester) to the control system regulating the activity of the progenitor cells (Huang and Herbert, 2006). In the presence of an intact rhythm, both drugs stimulate proliferation, but this is prevented if this rhythm is absent or flattened in Lister-Hooded (LH) rats. There is, therefore, an interaction between 5-HT and glucocorticoids in the dentate gyrus. Since both corticoids and 5-HT are highly responsive to external events—such as a stressful stimulus—these systems are a powerful way by which the external environment influences the rates of new neuron production in the adult hippocampus.

There are reports, in mice, of strain (i.e. genetic) differences in neurogenesis (Kempermann et al., 1997, 2006; Kim et al., 2008; Pozniak and Pleasure, 2006; Schauwecker, 2006). So far, there has been no exploration of similar differences in rats or, more importantly, whether these are dependent on equivalent differences in the activity of the hypothamalo-pituitary–adrenal (HPA) axis in either species. Neither is there information on whether sensitivity to 5-HT-acting drugs—such as fluoxetine—are strain-dependent or also related to strain-related differences in HPA activity. Since the actions of fluoxetine may have clinical as well as experimental interest, these questions need to be addressed.

In this paper we show that there are marked differences in basal rates of progenitor cell proliferation in two strains of male rats (Sprague–Dawley (SD) and LH); that this is associated with corresponding differences in basal levels of plasma corticosterone; that the progenitor cells in both strains respond to fluoxetine in an equivalent manner relative to their basal rates of proliferation but that this depends on the route of administration; that the response of SD rats to fluoxetine is also dependent on an intact diurnal corticoid rhythm; and, finally, that these strain differences are abolished by ADX and subsequent treatment with a standard replacement dose of corticosterone, pointing to a central role for the HPA axis in determining strain differences in neurogenesis.

## Experimental procedures

### Animals

All procedures were carried out under Home Office (UK) license. These regulations require the minimal use of animals, and adequate methods to reduce any suffering. Male LH or SD rats (Harlan, Oxon, UK) were used; they weighed around 200 g–250 g at the start of the experiment. Rats were housed in groups of three or five per cage in a controlled environment. Ambient temperature was maintained at 21 °C and humidity at 55% with *ad libitum* access to food and tap water (and normal saline for ADX animals). Animals were kept on reversed 12-h light/dark cycles (lights off at 10:00 h).

All surgical procedures were carried out under isoflurane anesthesia, and all animals received post-operative analgesia (buprenorphine). All animals were killed at CT (circadian time) 12–13 h (10:00–11:00 h).

### Experiment 1

#### Strain differences in proliferation rates and the effect of fluoxetine administration in LH and SD rats

Fluoxetine was given by two routes: daily i.p. injection and by osmotic minipump.

##### Experiment 1(a)

Two groups (*n*=5 per group) of each strain were given daily injections (i.p.) at CT12 (beginning of dark phase) of either fluoxetine (10 mg/kg/day) or vehicle (saline). After 14 days, the animals were terminally anesthetized with i.p. pentobarbitone, a blood sample was taken from the heart within 4 min of injection, and the brain was removed and frozen on dry ice. Subsequently, Ki67-labeled cells in the dentate gyrus were counted (see below).

##### Experiment 1(b)

Two groups (*n*=5 per group) of each strain were s.c. implanted with osmotic minipumps (Alzet 2ML2:5 μl/h) (Alzet Osmotic Pumps, Cupertino, CA, USA) filled either with fluoxetine dissolved in 25% propylene glycol) to deliver 10 mg/kg/day, or with vehicle. Minipumps were implanted dorsally. After 14 days, the animals were sacrificed as described above.

### Experiment 2

#### Effect of ADX with or without corticosterone replacement on basal cell proliferation levels in the dentate gyrus of adult LH and SD rats

Ten animals of each strain were ADX, and divided into two groups. One group received 5 mg/kg/day cort dissolved in sesame seed oil injected s.c. at the beginning of the dark phase (CT12), and the other was given vehicle alone. Treatment started on the day of ADX. All animals were killed after 7 days as described above.

### Experiment 3

#### Effect of clamping the diurnal corticosterone rhythm on progenitor cell response to fluoxetine in SD rats

Four groups (*n*=6 per group) of the SD strain were used. Two groups were implanted s.c. with osmotic minipumps (Alzet 2ML2: 5 μl/h) containing fluoxetine or vehicle as above. One of each of these two groups was also implanted s.c. with a corticosterone pellet (75 mg: Innovative Research, Sarasota, USA) to flatten the diurnal corticosterone rhythm (Leitch et al., 2003); the others received control (cholesterol) implants. Pellets and minipumps were implanted dorsally through the same incision. All animals were killed after 14 days as described above, plasma samples taken, and the brain processed for Ki67 (immunohistochemistry, IHC).

### Brain sections

Brains were stored for at least 24 h at −70 °C before sectioning. For each brain, coronal sections (20 μm; 1 in 6) were taken from the entire length of the dorsal hippocampus using a cryostat and mounted on a poly-lysine-coated microscope slides (BDH) and stored in −70 °C until required. The number of Ki67-labeled cells in the dentate gyrus was counted bilaterally on 12 sections per animal.

### Corticosterone assay

Total plasma corticosterone concentrations were measured by radioimmunoassay according to a validated procedure described previously (Chen and Herbert, 1995). The intra-assay coefficients of variation were: 5.1% for experiment 1, 6.2% for experiment 2 and 4.5% for experiment 3. The sensitivity of the assay was 0.98 ng/ml.

### IHC

Sections were first fixed for 5 min in 4% paraformaldehyde (pH 7.4, Fisher, Loughborough, UK) then rinsed twice with potassium phosphate buffer (KPBS). Sections were incubated in 0.01 M citric acid for 40 min at 98 °C, rinsed, and quenched with hydrogen peroxide for 10 min, rinsed with KPBS buffer before incubating and incubated overnight in KPBS containing 1% normal horse serum, 0.5% triton and mouse monoclonal antibody against Ki67 (1:100, Novocastra, Newcastle upon Tyne, UK). The sections were incubated with biotinylated secondary mouse IgG antibody and visualized with avidin–biotin–peroxidase complex, followed by the diaminobenzidine reaction (DAB). They were then dehydrated by passing through graded alcohols and incubated in Histoclear overnight, and coverslipped under mountant (DPX) for light microscopic analysis.

### Quantification

#### Proliferating cells

All slides were randomized and coded prior to quantitative analysis. Sections were examined using a 40× objective. Ki67-labeled cells were counted bilaterally in the dorsal hippocampus (one in six sections AP −2.30 to −4.52 from bregma on the Paxinos atlas: 12 sections per animal). Only cells on the internal border of the subgranular zone were included. The data shown are the mean count per section obtained from 12 sections per animal.

### Statistics

The mean±S.E.M. was determined for each group. Grouped data were analyzed using SPSS by analysis of variance (two-way ANOVA), after transformation of the data where necessary (significant Levene's test). Pairwise comparisons were made by Bonferroni post hoc tests or *t*-tests in experiments with only two groups.

## Results

### Strain differences in basal levels of progenitor cell proliferation and the effects of fluoxetine

#### I.p injections

**Fig. 1:**
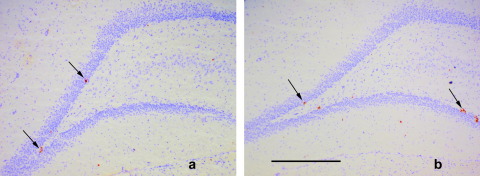
Photomicrographs of coronal sections (20 μm) through the dentate gyrus of (a) LH (b) SD strains of rats. Ki67-labeled cells are indicated by the arrows. Cresyl Violet background stain. Scale bar=500 μm.

One rat (SD, fluoxetine-treated) died. Control SD rats had around 60% more Ki67-labeled cells in the dentate gyrus than control LH rats (Fig. 1). There were significant effects of strain (two-way ANOVA: *F*_(1,15)_=4.8 (*P*>0.5) but not fluoxetine (*F*_(1.15)_=1.8, *P*>0.05) on the number of Ki67-labeled cells, and there was no strain×treatment interaction. Pairwise comparison of the two strains, irrespective of treatment, confirmed the significant difference between them (*t*=2.3 *P*=0.03). Plasma corticosterone showed reciprocal results. Levels were significantly lower in the SD strain (*F*_(1,13)_=13.5, *P*<0.01) but there was no treatment (fluoxetine) effect (*F*_(1,15)_=2.8, *P*>0.05) and no interaction. Pairwise comparison between strains was also significant (*t*=3.5, *P*=0.003).

#### Osmotic minipumps

**Fig. 2:**
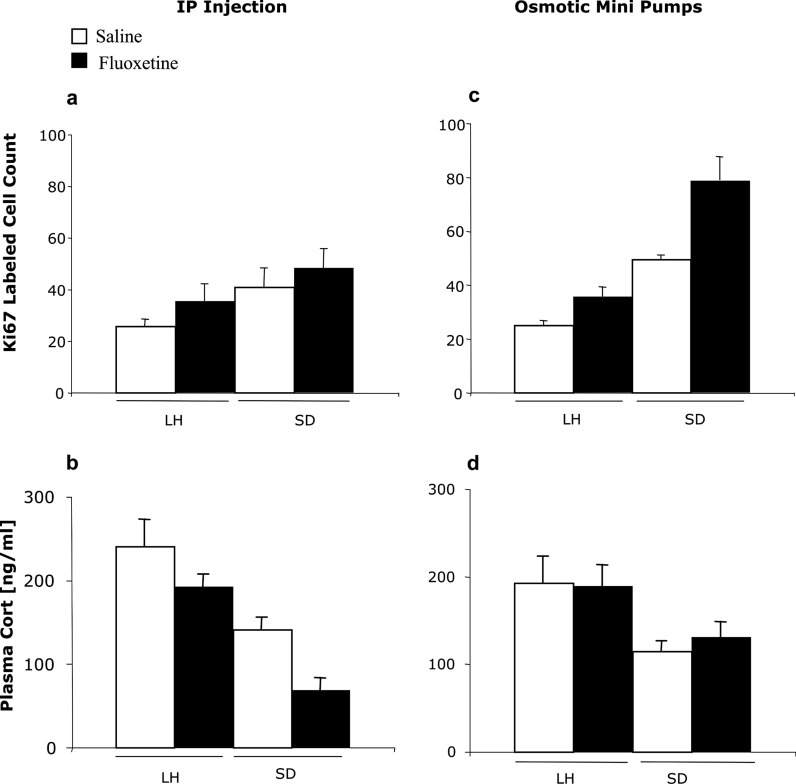
Number (mean per section±S.E.M.) of Ki67-labeled cells after either (a) i.p. saline (white bars) or fluoxetine (black bars) (c) saline- or fluoxetine-filled minipumps. (b, d) Plasma corticosterone levels in the same animals within 4 min of lethal injection of pentobarbital (at CT12). Significance levels (two-way ANOVA) given in text.

There were again strain differences in the basal expression of Ki67 in the dentate gyrus (SD>LH: two-way ANOVA: *F*_(1,16)_=79.6, *P*<0.001). Fluoxetine by this route increased the number of cells labeled with Ki67 (i.e. proliferating cells) (*F*_(1,16)_=23.2, *P*<0.001) but there was no interaction between strain and drug effects (*F*=0.39; *P*>0.05). The proportional increase in labeled cells following fluoxetine was about the same in both strains (65% vs. 62%) (Fig. 2c). Plasma corticosterone levels were significantly higher in LH than SD (*F*_(1,16)_=9.7, *P*<0.01). There was no change in plasma corticosterone levels following fluoxetine administration in either strain (*F*_(1,16)_=0.764, *P*>0.05) (Fig. 2d).

### Proliferating progenitor cells in the dentate gyrus following ADX and corticosterone replacement in LH and SD rats

**Fig. 3:**
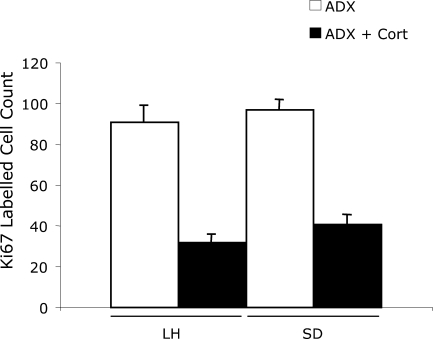
The effect of ADX and corticosterone replacement (5 mg/kg/day for 7 days) on cell proliferation (Ki67 counts) in the adult dentate gyrus of LH and SD strains. Significance levels (two-way ANOVA) given in text.

This experiment tested whether strain differences would remain after equalization of adrenal corticoid function. Two ADX rats from the LH strain were excluded because their plasma corticosterone was greater than 10 ng/ml. ADX increased levels of Ki67-labeled cells were high in both strains, compared with those in experiments 1 and 2. However, there was no longer any difference in Ki67 cell count between the two strains (*F*_(1,13)_=3.8, *P*>0.05). Daily corticosterone injections (5 mg/kg/day) significantly decreased the cell proliferation rate (*F*_(1,13)_=147.9, *P*<0.001) in both strains. The resulting values were, again, no different, and there was no strain×corticosterone interaction (*F*_(1,13)_=0.029, *P*>0.05) (Fig. 3).

### The effect of s.c. corticosterone on stimulation by fluoxetine of cell proliferation in SD

**Fig. 4:**
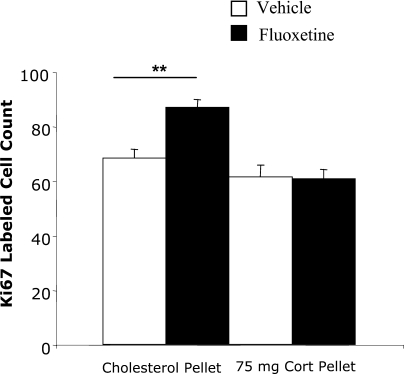
Effect of an s.c. pellet of corticosterone (75 mg) in intact SD rats on cell proliferation (Ki67 counts) in the dentate gyrus in control and fluoxetine-treated rats (osmotic minipumps). Results of two-way ANOVA given in text: significance levels of Bonferroni tests shown on fig: ** *P*<0.01.

We have already shown that flattening the diurnal corticosterone rhythm prevents the stimulating action of fluoxetine on progenitor cell proliferation in LH rats (Huang and Herbert, 2006). In view of the differences in corticosterone levels between the two strains, this experiment tested whether this also applies to SD rats. Corticosterone pellets implanted s.c. prevented the effect of fluoxetine given by osmotic minipump for 14 days on proliferation of progenitor cells in the SD strain. Fluoxetine increased Ki67 labeling (*F*_(1,16)_=7.8, *P*≤0.01), and corticosterone treatment also had a significant effect (*F*_(1,16)_=26.4, *P*<0.001). There was a marked interaction between fluoxetine and corticosterone treatments (*F*=8.9, *P*<0.01). Pairwise comparisons showed that while fluoxetine stimulated Ki67 in control-implanted rats (Bonferroni, *P*=0.003) this was not observed in corticosterone-treated ones (*P*>0.05) (Fig. 4).

## Discussion

This paper shows that there are marked basal differences in the proliferation rates (measured by Ki67 labeling, established as an indicator of mitosis: Pinnock et al. (2006)) of the progenitor cells in the dentate gyrus in two strains of rat, SD and LH, both of which have been used by different groups to study neurogenesis. There could be many mechanisms to account for these differences, including genetic factors that alter the niche environment in which these cells reside in the dentate gyrus. However, our results show clearly that strain differences in corticoids may account for much of this variation. SD rats had a much higher proliferation rate than LH, but also a significantly lower level of plasma corticosterone. Since we measured this at only one time point, we cannot provide further information on strain differences in the shape of the diurnal rhythm in this steroid. The association between corticosterone and mitosis rates in the dentate gyrus led us to suspect a functional relationship (Cameron and Gould, 1994; Huang and Herbert, 2006; Joels and Vreugdenhil, 1998; Wong and Herbert, 2005, 2006), and this proved to be the case. ADX, a procedure which removes endogenous corticoids, is well-known to increase basal rates of progenitor cell proliferation (Gould et al., 1992; Cameron and Gould, 1994). This occurred in both SD and LH: but the important point was that proliferation increased to the same absolute level in both strains, suggesting that the ‘ceiling’ was the same in both, and that control (intact) levels might be determined by strain differences in corticosterone. This was proved by giving both strains the same daily does of replacement corticosterone, which resulted in equal levels of proliferation. There is thus no strain difference in the sensitivity of the progenitor process to corticosterone, and it seems clear that it is the difference in basal corticosterone that determines those in neurogenesis between SD and LH male rats. The mechanisms for strain differences in the HPA axis remain to be explored, but were not an objective of the experiments reported here. Strain differences in neurogenesis may not be wholly dependent on those in the HPA axis. For example, 5-HT-related properties might play a part but this remains a topic for further study. It has been shown that aromatic amino acid decarboxylase (AADC), the enzyme responsible for converting the intermediate product of 5-HT (5-OH tryptophan) into its final form (5-HT) is higher in SD than LH in both brain stem and adrenal gland (Park et al., 1990), although AADC is not thought to be the key enzyme in the production of 5-HT.

Both strains also responded similarly to fluoxetine. We find that proliferation rates do not respond to daily i.p. injections of 10/mg/kg fluoxetine, and this confirms several other previous unpublished observations by our group. This is in contrast to those groups who report that i.p. injections are effective, though sometimes given twice daily or for a period more than 14 days (Manev et al., 2001) (Malberg et al., 2000). The reasons for these differences remain obscure. However, we consistently find that fluoxetine given by osmotic minipumps at this dose can stimulate proliferation rates. This seemed to be related to basal levels, since although the percentage increase was the same in both strains, the absolute levels of Ki67-labeled progenitor cells were higher in the SD strain following fluoxetine. It seems highly likely that these differences were also attributable to strain differences in plasma corticosterone; it is important to note that fluoxetine itself had no effect on corticosterone levels. The striking finding that flattening the diurnal rhythm in corticosterone prevents fluoxetine from increasing progenitor cell mitosis was reported in LH rats (Huang and Herbert, 2006), and here we confirm this finding and show that it applies also to SD rats, despite their lower levels of plasma corticosterone. It appears that an intact diurnal rhythm in corticosterone is a requirement for the effects of this SSRI on neurogenesis in both strains, suggesting a general phenomenon. There is still little definite information on the reasons why this rhythm is required for other controlling agents to access the process regulating progenitor cell proliferation, but studies on this are in progress.

The results reported here strengthen the role of glucocorticoids in the control of neurogenesis. They suggest that much of the lability and genetic differences associated with the rate and maturation of new neurons in the dentate gyrus of the hippocampus of adult rats can be ascribed to regulation by these steroids. Future studies directed toward better understanding of the functional implications of adult neurogenesis, as well as its potential clinical significance in, for example, the therapeutic response to anti-depressants such as fluoxetine (Drew and Hen, 2007; Herbert, 2008; Holick et al., 2007; Meshi et al., 2006; Santarelli et al., 2003), will need to take this into account.
